# Hexaaqua­zinc(II) dipicrate

**DOI:** 10.1107/S1600536808006624

**Published:** 2008-03-29

**Authors:** S. Natarajan, K. V. Vijitha, S. A. Martin Britto Dhas, J. Suresh, P. L. Nilantha Lakshman

**Affiliations:** a Department of Physics, Madurai Kamaraj University, Madurai 625 021, India; b Department of Physics, The Madura College, Madurai 625 011, India; c Department of Food Science and Technology, Faculty of Agriculture, University of Ruhuna, Mapalana, Kamburupitiya 81100, Sri Lanka

## Abstract

In the title compound, [Zn(H_2_O)_6_](C_6_H_2_N_3_O_7_)_2_, the Zn^II^ ion is located on an inversion center and is coordinated by six water mol­ecules in an octa­hedral geometry. The picrate anions have no coordination inter­actions with the Zn^II^ atom. The three nitro groups are twisted away from the attached benzene ring by19.8 (3), 6.5 (4) and 28.6 (3)°. There are numerous O—H⋯O hydrogen bonds in the crystal structure.

## Related literature

For related literature, see: Gartland *et al.* (1974 ▶); Herbstein *et al.* (1977 ▶); Liu *et al.* (2008 ▶); Maartmann-Moe (1969 ▶); Yang *et al.* (2001 ▶). 
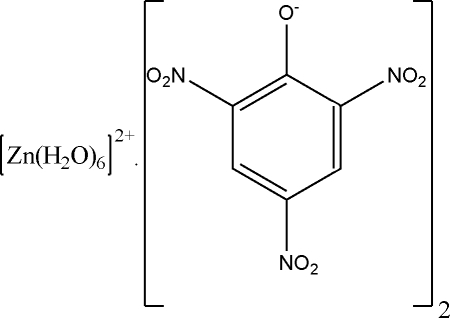


## Experimental

### 

#### Crystal data

[Zn(H_2_O)_6_](C_6_H_2_N_3_O_7_)_2_
*M* *_r_* = 629.68
Triclinic, 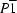

*a* = 7.8571 (4) Å
*b* = 8.3311 (6) Å
*c* = 8.9897 (7) Å
α = 89.8350 (11)°
β = 83.097 (1)°
γ = 72.8370 (9)°
*V* = 557.84 (7) Å^3^
*Z* = 1
Mo *K*α radiation
μ = 1.22 mm^−1^
*T* = 293 (2) K
0.13 × 0.11 × 0.10 mm

#### Data collection

Nonius MACH-3 diffractometer
Absorption correction: ψ scan (North *et al.*, 1968 ▶) *T*_min_ = 0.854, *T*_max_ = 0.886
2441 measured reflections
1971 independent reflections
1908 reflections with *I* > 2σ(*I*)
*R*_int_ = 0.006
2 standard reflections frequency: 60 min intensity decay: none

#### Refinement

*R*[*F* ^2^ > 2σ(*F* ^2^)] = 0.031
*wR*(*F* ^2^) = 0.087
*S* = 1.14
1971 reflections
196 parameters
4 restraints
H atoms treated by a mixture of independent and constrained refinement
Δρ_max_ = 0.42 e Å^−3^
Δρ_min_ = −0.71 e Å^−3^

### 

Data collection: *CAD-4 EXPRESS* (Enraf–Nonius, 1994 ▶); cell refinement: *CAD-4 EXPRESS*; data reduction: *XCAD4* (Harms & Wocadlo, 1996 ▶); program(s) used to solve structure: *SHELXS97* (Sheldrick, 2008 ▶); program(s) used to refine structure: *SHELXL97* (Sheldrick, 2008 ▶); molecular graphics: *PLATON* (Spek, 2003 ▶); software used to prepare material for publication: *SHELXL97*.

## Supplementary Material

Crystal structure: contains datablocks global, I. DOI: 10.1107/S1600536808006624/ci2563sup1.cif

Structure factors: contains datablocks I. DOI: 10.1107/S1600536808006624/ci2563Isup2.hkl

Additional supplementary materials:  crystallographic information; 3D view; checkCIF report

## Figures and Tables

**Table 1:** Hydrogen-bond geometry (Å, °)

*D*—H⋯*A*	*D*—H	H⋯*A*	*D*⋯*A*	*D*—H⋯*A*
O3*W*—H3*WB*⋯O1^i^	0.82 (2)	2.02 (2)	2.781 (2)	153 (3)
O3*W*—H3*WB*⋯O2^i^	0.82 (2)	2.38 (3)	2.972 (3)	130 (3)
O3*W*—H3*WA*⋯O2^ii^	0.84 (2)	2.07 (2)	2.880 (3)	164 (3)
O2*W*—H2*WB*⋯O3^ii^	0.82 (2)	2.48 (3)	3.083 (3)	131 (3)
O1*W*—H1*WA*⋯O6^iii^	0.83 (4)	1.99 (4)	2.799 (3)	164 (3)
O1*W*—H1*WB*⋯O1^iv^	0.80 (4)	1.99 (4)	2.705 (2)	149 (3)
O1*W*—H1*WB*⋯O6^iv^	0.80 (4)	2.24 (4)	2.839 (2)	132 (3)
O2*W*—H2*WB*⋯O4^v^	0.82 (2)	2.22 (3)	2.931 (3)	144 (3)
O2*W*—H2*WA*⋯O5^v^	0.82 (2)	2.57 (3)	3.097 (3)	123 (3)
O2*W*—H2*WA*⋯O7^vi^	0.82 (2)	2.46 (2)	3.223 (3)	154 (3)

Symmetry codes: (i) 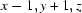; (ii) 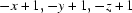; (iii) 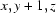; (iv) 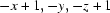; (v) 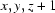; (vi) 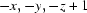.

**Fig. 1.**
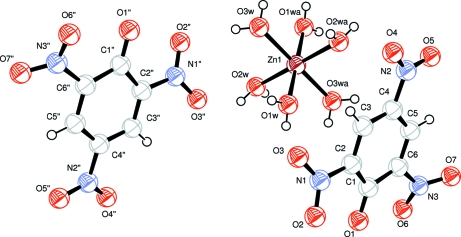
The molecular structure of the title compound, showing 30% probability displacement ellipsoids and the atom-numbering scheme. Atoms labeled with the suffix a and double prime (") are generated by the symmetry operations (-x, 1 - y, 1 - z) and (1 - x,1 - y, 1 - z), respectively.

**Fig. 2.**
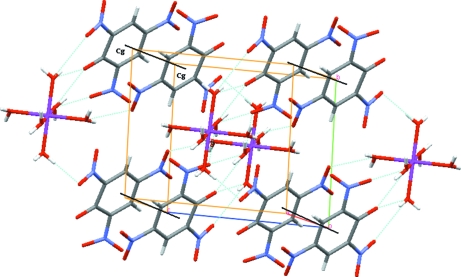
A packing diagram of the title compound. Dashed lines indicate π-π interactions.

## supplementary crystallographic information

### Comment

Picric acid forms salts with many organic and metallic cations (Gartland *et al.*, 1974). Picrates with various degrees of hydration are formed by metals (*e.g.* Li, Na), the alkaline earths (*e.g.* Cd, Hg) and various transition metals (*e.g.* Al, Y). Crystal structures have been reported for isomorphous NH_4_ and K picrates (Maartmann-Moe, 1969), thallium picrate (Herbstein *et al.*, 1977) and recently for manganese picrate (Liu *et al.*, 2008). The present work reports the crystal structure of the title compound, a zinc picrate. This work is part of a systematic investigation on the structures of the metal complexes of picric acid.

In the crystal structure of the title compound, each Zn^II^ ion is coordinated by the O atoms of six water molecules and not by the O atoms from the picrate anions. The Zn—O distances range from 2.0297 (16) to 2.1126 (17) Å. The coordination polyhedra around the Zn^II^ ion can be described as a distorted octahedron. The picrate anion adopts a keto form with a C1—O1 bond distance of 1.242 (3) Å; the C6—C1 [1.457 (3) Å] and C2—C1 [1.456 (3) Å] bond distances are longer than the other C—C bond lengths of the benzene ring. The three nitro groups are twisted out of the attached benzene ring by 19.8 (3)° [N1/O2/O3], 6.5 (4)° [N2/O4/O5] and 28.6 (3)° [N3/O6/O7]. The twisting of the nitro groups may be attributed to the O—H···O hydrogen bonding interactions taking place between water and picrate O atoms. The C2—C1—C6 bond angle of 111.20 (18)° is narrower than the corresponding angle in picric acid (116.4 (5)°; Yang *et al.*, 2001).

The packing of molecules is governed by large number of O—H···O hydrogen bonds (Table 1). π···π interactions are observed between the benzene rings of inversion related picrate ions, with a centroid to centroid distance of 3.6268 (11) Å (Fig. 2).

### Experimental

Colourless needle shaped single crystals of the title compound were grown from a saturated aqueous solution containing picric acid and zinc chloride in a 1:1 stoichiometric ratio.

### Refinement

O-bound H atoms were located in a difference Fourier map and their positional parameters were refined, with *U*_iso_(H) = 1.5*U*_eq_(O). Some of the O—H distances were restrained to 0.85 (2) Å. C-bound H atoms were placed at calculated positions and allowed to ride on their carrier atoms, with C—H = 0.93 Å, and *U*_iso_ = 1.2*U*_eq_(C).

### Crystal data

**Table:**

[Zn(H_2_O)_6_](C_6_H_2_N_3_O_7_)_2_	*Z* = 1
*M**_r_* = 629.68	*F*_000_ = 320
Triclinic, *P*1	*D*_x_ = 1.874 Mg m^−^^3^
Hall symbol: -P 1	Mo *K*α radiation λ = 0.71073 Å
*a* = 7.8571 (4) Å	Cell parameters from 25 reflections
*b* = 8.3311 (6) Å	θ = 2–25º
*c* = 8.9897 (7) Å	µ = 1.22 mm^−^^1^
α = 89.8350 (11)º	*T* = 293 (2) K
β = 83.097 (1)º	Needle, colourless
γ = 72.8370 (9)º	0.13 × 0.11 × 0.10 mm
*V* = 557.84 (7) Å^3^	

### Data collection

**Table:**

Nonius MACH-3 diffractometer	*R*_int_ = 0.006
Radiation source: fine-focus sealed tube	θ_max_ = 25.0º
Monochromator: graphite	θ_min_ = 2.3º
*T* = 293(2) K	*h* = −1→9
ω–2θ scans	*k* = −9→9
Absorption correction: ψ scan(North *et al.*, 1968)	*l* = −10→10
*T*_min_ = 0.854, *T*_max_ = 0.886	2 standard reflections
2441 measured reflections	every 60 min
1971 independent reflections	intensity decay: none
1908 reflections with *I* > 2σ(*I*)	

### Refinement

**Table:**

Refinement on *F*^2^	Secondary atom site location: difference Fourier map
Least-squares matrix: full	Hydrogen site location: inferred from neighbouring sites
*R*[*F*^2^ > 2σ(*F*^2^)] = 0.031	H atoms treated by a mixture of independent and constrained refinement
*wR*(*F*^2^) = 0.087	*w* = 1/[σ^2^(*F*_o_^2^) + (0.0559*P*)^2^ + 0.2704*P*] where *P* = (*F*_o_^2^ + 2*F*_c_^2^)/3
*S* = 1.14	(Δ/σ)_max_ = 0.001
1971 reflections	Δρ_max_ = 0.43 e Å^−^^3^
196 parameters	Δρ_min_ = −0.71 e Å^−^^3^
4 restraints	Extinction correction: none
Primary atom site location: structure-invariant direct methods	

### Special details

**Table:**

Geometry. All e.s.d.'s (except the e.s.d. in the dihedral angle between two l.s. planes) are estimated using the full covariance matrix. The cell e.s.d.'s are taken into account individually in the estimation of e.s.d.'s in distances, angles and torsion angles; correlations between e.s.d.'s in cell parameters are only used when they are defined by crystal symmetry. An approximate (isotropic) treatment of cell e.s.d.'s is used for estimating e.s.d.'s involving l.s. planes.
Refinement. Refinement of *F*^2^ against ALL reflections. The weighted *R*-factor *wR* and goodness of fit *S* are based on *F*^2^, conventional *R*-factors *R* are based on *F*, with *F* set to zero for negative *F*^2^. The threshold expression of *F*^2^ > σ(*F*^2^) is used only for calculating *R*-factors(gt) *etc*. and is not relevant to the choice of reflections for refinement. *R*-factors based on *F*^2^ are statistically about twice as large as those based on *F*, and *R*- factors based on ALL data will be even larger.

### Fractional atomic coordinates and isotropic or equivalent isotropic displacement parameters (Å^2^)

**Table:**

	*x*	*y*	*z*	*U*_iso_*/*U*_eq_	
Zn1	0.0000	0.5000	0.5000	0.02629 (14)	
O1W	0.2724 (2)	0.4246 (2)	0.4648 (2)	0.0388 (4)	
H1WA	0.325 (5)	0.498 (5)	0.458 (4)	0.058*	
H1WB	0.328 (5)	0.351 (5)	0.513 (4)	0.058*	
O2W	−0.0055 (3)	0.5054 (2)	0.73549 (19)	0.0421 (4)	
H2WB	0.085 (3)	0.504 (5)	0.774 (4)	0.063*	
H2WA	−0.055 (5)	0.439 (4)	0.775 (4)	0.063*	
O3W	−0.0111 (2)	0.7546 (2)	0.4830 (2)	0.0404 (4)	
H3WA	0.014 (5)	0.812 (4)	0.549 (3)	0.061*	
H3WB	−0.104 (3)	0.817 (4)	0.455 (4)	0.061*	
O1	0.6551 (2)	−0.1387 (2)	0.37983 (19)	0.0378 (4)	
O2	0.8538 (3)	0.0707 (2)	0.3227 (3)	0.0595 (6)	
O3	0.7109 (3)	0.3187 (3)	0.2651 (3)	0.0595 (6)	
O4	0.2551 (3)	0.3894 (2)	−0.0507 (2)	0.0517 (5)	
O5	0.0834 (3)	0.2284 (3)	−0.0310 (2)	0.0547 (5)	
O6	0.4083 (3)	−0.3049 (3)	0.3983 (2)	0.0514 (5)	
O7	0.2971 (3)	−0.3042 (3)	0.1909 (2)	0.0571 (5)	
N1	0.7226 (3)	0.1697 (2)	0.2785 (2)	0.0353 (4)	
N2	0.2129 (3)	0.2680 (3)	0.0013 (2)	0.0380 (5)	
N3	0.3679 (3)	−0.2413 (3)	0.2789 (2)	0.0350 (4)	
C1	0.5553 (3)	−0.0462 (3)	0.2971 (2)	0.0265 (4)	
C2	0.5767 (3)	0.1112 (3)	0.2393 (2)	0.0275 (4)	
C3	0.4649 (3)	0.2134 (3)	0.1486 (2)	0.0303 (5)	
H3	0.4833	0.3143	0.1178	0.036*	
C4	0.3249 (3)	0.1648 (3)	0.1037 (2)	0.0302 (5)	
C5	0.2933 (3)	0.0163 (3)	0.1492 (2)	0.0304 (5)	
H5	0.1993	−0.0154	0.1173	0.037*	
C6	0.4035 (3)	−0.0836 (3)	0.2424 (2)	0.0278 (4)	

### Atomic displacement parameters (Å^2^)

**Table:**

	*U*^11^	*U*^22^	*U*^33^	*U*^12^	*U*^13^	*U*^23^
Zn1	0.0234 (2)	0.0250 (2)	0.0316 (2)	−0.00531 (14)	−0.01323 (14)	0.00959 (13)
O1W	0.0258 (8)	0.0363 (9)	0.0567 (11)	−0.0088 (7)	−0.0161 (7)	0.0218 (8)
O2W	0.0460 (11)	0.0465 (10)	0.0327 (9)	−0.0074 (8)	−0.0172 (8)	0.0094 (7)
O3W	0.0395 (10)	0.0272 (9)	0.0598 (11)	−0.0089 (7)	−0.0290 (8)	0.0117 (8)
O1	0.0341 (9)	0.0369 (9)	0.0472 (10)	−0.0110 (7)	−0.0233 (7)	0.0217 (7)
O2	0.0513 (12)	0.0418 (11)	0.0980 (16)	−0.0184 (9)	−0.0471 (11)	0.0189 (10)
O3	0.0736 (14)	0.0418 (11)	0.0806 (15)	−0.0325 (10)	−0.0394 (12)	0.0258 (10)
O4	0.0565 (12)	0.0461 (11)	0.0510 (11)	−0.0064 (9)	−0.0245 (9)	0.0264 (9)
O5	0.0469 (11)	0.0612 (12)	0.0593 (12)	−0.0097 (10)	−0.0363 (10)	0.0197 (10)
O6	0.0471 (11)	0.0599 (12)	0.0616 (12)	−0.0293 (9)	−0.0297 (9)	0.0399 (10)
O7	0.0721 (14)	0.0606 (13)	0.0585 (12)	−0.0428 (11)	−0.0275 (11)	0.0166 (10)
N1	0.0406 (11)	0.0325 (10)	0.0379 (10)	−0.0134 (9)	−0.0182 (9)	0.0084 (8)
N2	0.0372 (11)	0.0385 (11)	0.0298 (10)	0.0053 (9)	−0.0136 (8)	0.0075 (8)
N3	0.0278 (10)	0.0398 (11)	0.0412 (11)	−0.0128 (8)	−0.0119 (8)	0.0136 (9)
C1	0.0245 (10)	0.0266 (10)	0.0260 (10)	−0.0023 (8)	−0.0079 (8)	0.0063 (8)
C2	0.0292 (11)	0.0272 (10)	0.0267 (10)	−0.0065 (9)	−0.0105 (8)	0.0047 (8)
C3	0.0359 (12)	0.0257 (10)	0.0262 (10)	−0.0028 (9)	−0.0083 (9)	0.0058 (8)
C4	0.0292 (11)	0.0316 (11)	0.0244 (10)	0.0022 (9)	−0.0107 (8)	0.0065 (8)
C5	0.0232 (10)	0.0387 (12)	0.0270 (10)	−0.0034 (9)	−0.0087 (8)	0.0046 (9)
C6	0.0257 (10)	0.0300 (11)	0.0272 (10)	−0.0062 (8)	−0.0069 (8)	0.0083 (8)

### Geometric parameters (Å, °)

**Table:**

Zn1—O1W^i^	2.0297 (16)	O4—N2	1.228 (3)
Zn1—O1W	2.0297 (16)	O5—N2	1.224 (3)
Zn1—O3W^i^	2.1025 (16)	O6—N3	1.230 (3)
Zn1—O3W	2.1025 (16)	O7—N3	1.221 (3)
Zn1—O2W^i^	2.1126 (17)	N1—C2	1.451 (3)
Zn1—O2W	2.1126 (17)	N2—C4	1.451 (3)
O1W—H1WA	0.83 (4)	N3—C6	1.451 (3)
O1W—H1WB	0.80 (4)	C1—C2	1.456 (3)
O2W—H2WB	0.822 (18)	C1—C6	1.457 (3)
O2W—H2WA	0.825 (18)	C2—C3	1.374 (3)
O3W—H3WA	0.837 (18)	C3—C4	1.381 (3)
O3W—H3WB	0.824 (19)	C3—H3	0.93
O1—C1	1.242 (3)	C4—C5	1.383 (3)
O2—N1	1.223 (3)	C5—C6	1.374 (3)
O3—N1	1.224 (3)	C5—H5	0.93
			
O1W^i^—Zn1—O1W	180.0	O3—N1—C2	118.43 (19)
O1W^i^—Zn1—O3W^i^	92.05 (7)	O5—N2—O4	123.3 (2)
O1W—Zn1—O3W^i^	87.95 (7)	O5—N2—C4	118.6 (2)
O1W^i^—Zn1—O3W	87.95 (7)	O4—N2—C4	118.1 (2)
O1W—Zn1—O3W	92.05 (7)	O7—N3—O6	122.8 (2)
O3W^i^—Zn1—O3W	180.0	O7—N3—C6	118.70 (19)
O1W^i^—Zn1—O2W^i^	92.82 (8)	O6—N3—C6	118.5 (2)
O1W—Zn1—O2W^i^	87.18 (8)	O1—C1—C2	124.7 (2)
O3W^i^—Zn1—O2W^i^	93.38 (8)	O1—C1—C6	124.1 (2)
O3W—Zn1—O2W^i^	86.62 (8)	C2—C1—C6	111.20 (18)
O1W^i^—Zn1—O2W	87.18 (8)	C3—C2—N1	115.76 (19)
O1W—Zn1—O2W	92.82 (8)	C3—C2—C1	124.3 (2)
O3W^i^—Zn1—O2W	86.62 (8)	N1—C2—C1	119.89 (18)
O3W—Zn1—O2W	93.38 (8)	C2—C3—C4	119.3 (2)
O2W^i^—Zn1—O2W	180.0	C2—C3—H3	120.3
Zn1—O1W—H1WA	118 (2)	C4—C3—H3	120.3
Zn1—O1W—H1WB	120 (3)	C3—C4—C5	121.54 (19)
H1WA—O1W—H1WB	107 (3)	C3—C4—N2	119.3 (2)
Zn1—O2W—H2WB	121 (3)	C5—C4—N2	119.1 (2)
Zn1—O2W—H2WA	111 (3)	C6—C5—C4	118.8 (2)
H2WB—O2W—H2WA	112 (4)	C6—C5—H5	120.6
Zn1—O3W—H3WA	125 (3)	C4—C5—H5	120.6
Zn1—O3W—H3WB	116 (2)	C5—C6—N3	115.6 (2)
H3WA—O3W—H3WB	104 (3)	C5—C6—C1	124.8 (2)
O2—N1—O3	121.6 (2)	N3—C6—C1	119.51 (18)
O2—N1—C2	119.98 (19)		
			
O2—N1—C2—C3	−160.3 (2)	O5—N2—C4—C5	6.7 (3)
O3—N1—C2—C3	19.3 (3)	O4—N2—C4—C5	−172.1 (2)
O2—N1—C2—C1	20.3 (3)	C3—C4—C5—C6	0.6 (3)
O3—N1—C2—C1	−160.1 (2)	N2—C4—C5—C6	177.90 (19)
O1—C1—C2—C3	−179.5 (2)	C4—C5—C6—N3	−176.93 (19)
C6—C1—C2—C3	1.8 (3)	C4—C5—C6—C1	−0.7 (3)
O1—C1—C2—N1	−0.2 (3)	O7—N3—C6—C5	26.2 (3)
C6—C1—C2—N1	−178.87 (19)	O6—N3—C6—C5	−152.9 (2)
N1—C2—C3—C4	178.65 (19)	O7—N3—C6—C1	−150.2 (2)
C1—C2—C3—C4	−2.0 (3)	O6—N3—C6—C1	30.7 (3)
C2—C3—C4—C5	0.7 (3)	O1—C1—C6—C5	−179.1 (2)
C2—C3—C4—N2	−176.59 (19)	C2—C1—C6—C5	−0.4 (3)
O5—N2—C4—C3	−176.0 (2)	O1—C1—C6—N3	−3.0 (3)
O4—N2—C4—C3	5.2 (3)	C2—C1—C6—N3	175.65 (19)

Symmetry codes: (i) −*x*, −*y*+1, −*z*+1.

### Hydrogen-bond geometry (Å, °)

**Table:**

*D*—H···*A*	*D*—H	H···*A*	*D*···*A*	*D*—H···*A*
O3W—H3WB···O1^ii^	0.82 (2)	2.02 (2)	2.781 (2)	153 (3)
O3W—H3WB···O2^ii^	0.82 (2)	2.38 (3)	2.972 (3)	130 (3)
O3W—H3WA···O2^iii^	0.84 (2)	2.07 (2)	2.880 (3)	164 (3)
O2W—H2WB···O3^iii^	0.82 (2)	2.48 (3)	3.083 (3)	131 (3)
O1W—H1WA···O6^iv^	0.83 (4)	1.99 (4)	2.799 (3)	164 (3)
O1W—H1WB···O1^v^	0.80 (4)	1.99 (4)	2.705 (2)	149 (3)
O1W—H1WB···O6^v^	0.80 (4)	2.24 (4)	2.839 (2)	132 (3)
O2W—H2WB···O4^vi^	0.82 (2)	2.22 (3)	2.931 (3)	144 (3)
O2W—H2WA···O5^vi^	0.82 (2)	2.57 (3)	3.097 (3)	123 (3)
O2W—H2WA···O7^vii^	0.82 (2)	2.46 (2)	3.223 (3)	154 (3)

Symmetry codes: (ii) *x*−1, *y*+1, *z*; (iii) −*x*+1, −*y*+1, −*z*+1; (iv) *x*, *y*+1, *z*; (v) −*x*+1, −*y*, −*z*+1; (vi) *x*, *y*, *z*+1; (vii) −*x*, −*y*, −*z*+1.
